# Amputation of the Cervix Uteri

**Published:** 1879-05

**Authors:** W. S. Caldwell

**Affiliations:** London


Amputation of the Cervix Uteri.—Of the various modes of doing this operation, Prof. Langenbeck prefers the knife or the scissors. The écraseur, he says, should *never* be used for this purpose. He relates a case that occurred in his clinic, when Prof. Bilroth was his assistant, in which, although he applied the instrument with great care, and kept his finger on the chain during the whole operation, Prof. B. tightening the screw, a portion of the bladder was included in the incision, and the patient died two days later of peritonitis The objection that Prof. Langenbeck has given to galvano-cautery in this operation is that you can never be positively sure *just* how much tissue you are removing.

On the other hand, since I have been in London I have seen Prof. Barnes amputate the cervix twice with the écraseur. He used the instrument without a speculum, being guided wholly by the index finger of eachhand, which he kept in contact with the chain.

After the cervix is removed, he applies the thermo-cautery, to arrest any hæmorrhage that may exist. This, he tells me, is his *uniform* mode of doing this operation, and that he is perfectly satisfied with his results. Prof. Barnes brought out a patient and laid her on the operating table, telling me as he did so that he was going to show me the American operation (Emmet's).

But on examining the cervix, and seeing it very red and congested, he concluded that he would wait a few days until the congestion in the parts subsided. In fact, he feared the hæmorrhage that would follow. I could but contrast his timidity with the confidence with which some German operators would have attacked these parts with the knife without the least fear or hesitation.

I have seen the amputation of the cervix done with the scalpel about forty times during my stay in Berlin, and not in a single instance have I seen any serious trouble in arresting the bleeding. Dr. A. Martin, of this city, read a paper last year before the gynæcological section of the German Medical Society, on "The

Therapeutics of Chronic Metritis," in which, after going on to state that all as medications, both local and constitutional, had proved of no avail in the management of the disease, he had been induced to resort to an operative procedure for its cure, which operation consisted in an amputation of the cervix uteri.

At that date (August last) he had done the operation 109 times. Of these patients, 72 suffered from a simple chronic metritis, one-half of which number he had had under observation and treatment for many months without the least improvement in their condition. Twenty-six were performed for a cancerous condition of the cervix, and eight for a local hypertroph of one or both lips of the neck of the womb. Of the cases, only two died as a direct result of the operation. One of these was very anæmic from loss of blood before the operation, and died of septicæmia soon after. The other proved fatal from the supervention of typhoid fever.

His mode of doing this operation is to cut away a portion somewhat funnel-shaped, the base below and the apex above, of from 3 to 5 centimeters in length, depending upon the length of the uterus, as measured by the sound before he operates.

I have followed Dr. Martin carefully for nearly four months, and have seen him and assisted him at this operation more than thirty times; and while I am perfectly sure that no écraseur nor galvano-cautery can do this operation so well as does the knife, I am sorry to be compelled to believe that, like some other of our noted gynæcologists, he is bent on doing "*his*" operation as often as possible, and so submits many a poor woman to this treatment, who might be relieved by a more simple procedure.

Unfortunately, like many another celebrated operator, he is inclined, I fear, to keep in the background as much as possible the accidents that follow his treatment.

I saw a woman in the service of another gynæcologist of Berlin, operated on for complete occlusion of the os uteri and hæmatometra, that followed an amputation of the cervix by Dr. Martin.

When I related this case to Dr. M., he said that this patient was one of two who had had secondary hæmorrhage following the operation, and he attributed this bad result in her case to the extra stitches that he had to put in, in order to stop the bleeding, as well as her having left his service and passed out from under his observation sooner than she should have done. Prof. Schröder's mode of amputating the cervix, and which Prof. Langenbeck recommends, is, in my mind, at once the most simple and easy of execution of any of the numerous modes of doing this operation that I have seen tried. Dr. Martin, however, objects to it because the resulting stump is somewhat flat, while when done after his plan (Dr. Martin's) the stump is conical in shape. Prof. Schröder's reply to this is, that nature has to re-shape the parts after any cutting procedure of this kind, and that in the end his operation leaves as natural cervix as any other. To do this operation Prof. Schröder places his patient on her back, her thighs flexed upon the abdomen and the legs upon the thighs. (I have never seen a German operator place a woman in Sims' position).

A Simm's speculum is next introduced, and the perineum pressed well down. A Hegar's irrigator containing a 5 per cent solution of carbolic acid is now made to throw a constant stream of this fluid upon the cervix uteri during the entire operation. In fact, Prof. Schröder claims to make this operation strictly antiseptically, substituting the fluid carbolized solution for the spray. A pair of scissors are now introduced into the os uteri, and the os incised as high up as the vaginal insertion on each side. The cervix is thus divided into two flaps, and while an assistant pulls down and steadies the womb by means of a pair of forceps made fast to one flap, the surgeon seizes the other and amputates it readily with the knife, cutting, of course, from below upwards, so that you have left after the amputation a somewhat long flap-shaped mucous membrane to cover the stump.

By means of a curved needle, armed with a heavy silk ligature, two sutures are now passed from the vaginal mucous surface of the stump into the os. These sutures are each placed 1 centimeter from the median line at their point of entry into the vaginal walls. They are now tied tightly, and serve to render the os uteri patulous, as well as to pull down and hold the womb firmly, while the other lip is cut away in the same manner. From three to four stitches are next applied on each side of these central ones, and the operation is completed. The vagina is now tamponed well with salicylated cotton, which seals up the wound and makes the whole procedure antiseptic.

With the uterus thus firmly held, and with a needle curved to a semi-circle, and armed with a silk thread, there is but little difficulty in passing a suture through the muscular walls of the uterine stump in such a manner that when it is tightly tied it will include any bleeding vessel and effectually arrest any hæmorrhage.

German operators do not use silver wire for sutures, either for this operation or Emmet's, as they claim that the silk answers every purpose, and is much easier to apply and remove.

Prof. Langenbeck lays down one very emphatic rule as to the use of styptics to arrest hæmorrhage where operations have been done in the vagina, and where the probabilities are that the peritoneum has been encroached upon. *Never*, he says, use preparations of *iron* for this purpose. He relates several cases where he used a solution of iron, and after his patients died of peritonitis he found that the walls of the peritoneum had sloughed away from the effect of his styptic. He believes that even a moderately strong solution of iron will cause a necrosis of any serous membrane to which it is applied.

Prof. Freund, late of Breslau, but now called to Strasburg to fill the place of Prof. Gusserow, appointed as Professor of Midwifery in the University of Berlin, spent a month in the latter city this winter. As the reader perhaps knows, Prof. Freund is the father of the revised method of extirpation of the entire uterus through the abdominal walls.

I saw him operate on a case for Dr. Schröder just before I left Berlin. The patient was alive four days after the operation, but her recovery was considered doubtful. I saw him begin another operation of the same kind for Dr. Martin, but after making his abdominal incision, he found the pelvic glands so invaded by cancerous infiltration that he would not attempt the removal of the uterus.

He closed up the wound, and Dr. Martin then performed his amputation of the cervix uteri as a palliatory expedient. This poor woman had a good deal of constitutional disturbance, but was convalescing the last time I saw her, ten days after the operation.

Prof. Freund has done this operation of the total extirpation of the womb 12 times and has had 7 recoveries, while in Dr. West's tabulated 25 cases there occurred 23 deaths.

The enucleation of the womb entire through the vaginal walls, of which a lately published case is reported by Prof. Lane (I believe) of San Francisco, Prof. Langenbeck considers an anatomical *impossibility*.

He says, that while you may with considerable facility separate the *cervical* portion of the womb from its attachment, that when you reach the *body* of that organ, you cannot separate its muscular tissues from its peritoneal coating without the greatest difficulty, even when you have the uterus removed from the body and dissect with the greatest care, and as an operative procedure upon the living body he does not think its accomplishment possible.

Tracheotomy.—A child six years old was brought into Prof. Langenbeck's clinic, and being placed upon the table. He said:

"Gentlemen, you would scacrely imagine on looking at this little boy and seeing how little his respiration is embarrassed, that I am about to open his trachea."

The patient was suffering from diphtheria, and had twenty-four hours previously developed a croupy cough. Prof. L. laid it down as a rule, to which there should be no exception, that in the management of these cases you should operate before signs of extreme dyspnœa and blood poisoning come on. Waiting will do no good, for with an almost absolute certainty tracheotomy will be necessary, and the longer you wait the less are your chances of success. The operation of itself is not dangerous unless done when the patient is in extremis. Since 1870 he has done this operation 700 times in his clinic. In some years he has saved as high as 40 per cent of his cases operated on, and in others only 10 per cent.

He was not able to explain why there should be this great difference in the mortality of his cases in different years; but one fact he did know, and that was that since he had given out word to the physicians of Berlin that they should send their diphtheritic patient earlier for this operation, his success has been much better.

In the City hospital of Berlin diphtheritic patients are kept in a ward adjoining that devoted to the chronic diseases of women. On making one of my evening visits to this institution with Dr. Alberts, physician in charge, he showed me a boy ten years old who had just been brought in with this disease, and upon whom he proposed to perform the operation of tracheotomy if the case did not improve after one hour.

I administered the chloroform, and the doctor cut down through the skin and underlying fascia, and with great care separated, as he thought, the two lobes of the thyroid gland with the handle of his scalpel, and finally reaching what he thought to be the trachea, made an incision into it. No air escaped, though he pulled the edges of the wound open with a pair of forceps. The true condition of things now flashed over him in an instant. He had gone outside of and behind the trachea, and had opened into the œsophagus. After waiting a few minutes and expressing his great horror at what he termed his *extreme stupidity*, he opened the trachea and put in the tube. With even this mishap this case did well as far as the operation was concerned, though the patient died five days later from extention of the diphtheritic process into the bronchi. The boy was not given any nourishment by the mouth for 24 hours, but after that time he was given fluid nourishment, which passed into the stomach without hindrance.

W. S. Caldwell.

London, March 3d, 1879.

